# Infants learn better from left to right: a directional bias in infants’ sequence learning

**DOI:** 10.1038/s41598-017-02466-w

**Published:** 2017-05-26

**Authors:** Hermann Bulf, Maria Dolores de Hevia, Valeria Gariboldi, Viola Macchi Cassia

**Affiliations:** 1 Department of Psychology, University of Milano-Bicocca, Piazza Ateneo Nuovo 1, 20126 Milan, Italy; 2 NeuroMi, Milan Center for Neuroscience, Milan, Italy; 3 Université Paris Descartes, Sorbonne Paris Cité, Paris, France; 4 CNRS UMR 8242, Laboratoire Psychologie de la Perception, Paris, France

## Abstract

A wealth of studies show that human adults map ordered information onto a directional spatial continuum. We asked whether mapping ordinal information into a directional space constitutes an early predisposition, already functional prior to the acquisition of symbolic knowledge and language. While it is known that preverbal infants represent numerical order along a left-to-right spatial continuum, no studies have investigated yet whether infants, like adults, organize any kind of ordinal information onto a directional space. We investigated whether 7-month-olds’ ability to learn high-order rule-like patterns from visual sequences of geometric shapes was affected by the spatial orientation of the sequences (left-to-right vs. right-to-left). Results showed that infants readily learn rule-like patterns when visual sequences were presented from left to right, but not when presented from right to left. This result provides evidence that spatial orientation critically determines preverbal infants’ ability to perceive and learn ordered information in visual sequences, opening to the idea that a left-to-right spatially organized mental representation of ordered dimensions might be rooted in biologically-determined constraints on human brain development.

## Introduction

Serial order processing refers to the understanding of specific spatiotemporal relations between the elements in a sequence, and concerns the ways we code, store, represent and reproduce the order of these elements (e.g., refs 1 and 2). As processing of ordinal information is fundamental to operate within a spatiotemporally bounded environment, many studies have investigated its ontogenetic and phylogenetic roots. The ability to learn sequences and their ordinal structure has been found in few-month-old infants (e.g., ref. 3), and in non-human animals (e.g., refs 4–6), suggesting that the ability to process serial order is shared among species^7^, and builds on a domain-general core mechanism that is functional prior to the acquisition of symbolic knowledge and language^8^.

Evidence is mounting showing that the coding of ordinal information may co-opt a spatially organized mental representation. Human adults exploit a spatially oriented horizontal continuum to represent not only information learnt in a conventional fixed order, like numbers (e.g., refs 9–11), months of the year and days of the week (e.g., ref. 12), letters of the alphabet (e.g., ref. 13), or temporal events (e.g., ref. 14), but also non-ordered newly-learnt information, such as lists of unrelated words (e.g., refs 15 and 16). Together, this evidence suggests that adults’ privileged way to mentally organize (both over-learnt and newly-learnt) serially ordered information takes the form of an oriented spatial representation. To account for this evidence, it has been proposed that spatial-ordinal associations are temporarily constructed in working memory during task execution^16^, whereby ordered elements become spatially coded according to their ordinal position in a sequence^17, 18^.

As the directionality of the temporary associations of ordinal positions and space is modulated by culture (e.g., refs 19 and 20), it has been hypothesized that scanning habits associated to reading and writing practices are critical in establishing preferential directional scanning of the external space and its internal representation^21^. Within this view, the left-to-right direction of the spatial-ordinal association found in Western cultures (as they present left-to-right directional writing/reading practices) would emerge during development as a consequence of the impact of culturally-driven routines^22^. However, if spatial coding of ordinal information does not reflect long-term memory representations of intrinsically-ordered dimensions, but it is constructed online whenever elements’ order is critical to the task, spatial-ordinal mappings might be involved during learning of serially ordered information even in early stages of development, when infants lack symbolic knowledge, and the ability to store and retain information for long time is still limited.

The current study tested this possibility by investigating whether directional spatial information (left-to-right versus right-to-left) modulates preverbal infants’ learning of ordered sequences. Indeed, it has been recently shown that 7- to 9-month-old infants’ learning of ordinal relations embedded in numerical sequences is critically impacted by the direction with which the sequences are provided^23, 24^. For example, 7-month-olds are able to discriminate inversion in numerical ordinal direction (increasing vs. decreasing) when sequences of numerical displays are presented in the center of the screen^25^ or from left to right^24^, but not when presented from right to left^24^. This finding has been taken as evidence that symbolic knowledge and formal education are not critical in establishing a preferential directionality of the association between numerical order and space^26, 27^. However, to date no evidence has been provided as to whether preverbal infants, like adults, use space to represent any type of ordered information, especially when order is not an intrinsic feature but one that is coded and learned during task execution.

Here we investigated whether linking each element’s position to distinct adjacent spatial positions may play a pivotal role in the ability to construct, maintain, and retrieve ordinal information from the earliest stages of development. Alternatively, this phenomenon may represent a learned strategy mediated by language, and therefore may be acquired by humans across development. We therefore tested the impact of directional spatial information on preverbal infants’ capacity to learn and abstract high-order rule-like patterns defined by the ordinal position of the items within visual sequences that are not intrinsically ordered.

Rule learning refers to the ability to detect high-order repetition-based rules (i.e., ABB, AAB, ABA) from a sequence of elements and to generalize them to new items^28^. For instance, it was demonstrated that 7 month old infants familiarized to sequences of syllables that followed a particular ABB rule-like grammar (e.g., la ta ta, gai mu mu) are able to extract the rule embedded in the speech sequence, generalize it to novel stimuli, and discriminate it from other, similar patterns^28^. This implies going beyond the surface form of patterns, by abstracting the structure of the sequence and generalize it to new sequences. In spite of the original claim that infants’ rule-learning abilities are confined to speech processing^29^, research has shown that rule learning is a domain-general mechanism as it occurs for visual stimuli as well both in infants^30–32^ and animals^33^. Specifically, 7-month-olds extract and learn ABB rule-like patterns, but not ABA patterns, from visual sequences composed of geometric shapes^31^, while they readily represent both patterns when presented with sequences composed of real images of faces^30^ or dogs^32^. As the ABA rule is considered more complex than the ABB rule, as it involves non-adjacent repetitions of the A element, 7-month-olds’ success in learning the ABA rule in some studies has been accounted for by proposing that stimulus familiarity may boost infants’ rule learning abilities. However, another critical factor to consider is the spatiotemporal layout within which the visual sequences were delivered to infants. When infants failed to learn the ABA rule, stimuli were centered on the screen^31^, whereas when infants succeeded to learn the ABA rule, stimuli were spatiotemporally presented from left to right^30, 32^. Therefore, it is possible that directional spatial information had boosted infants’ sensitivity to the ordinal positions of the items, thus enhancing their ability to learn the rule embedded in the sequences.

**Figure 1:**
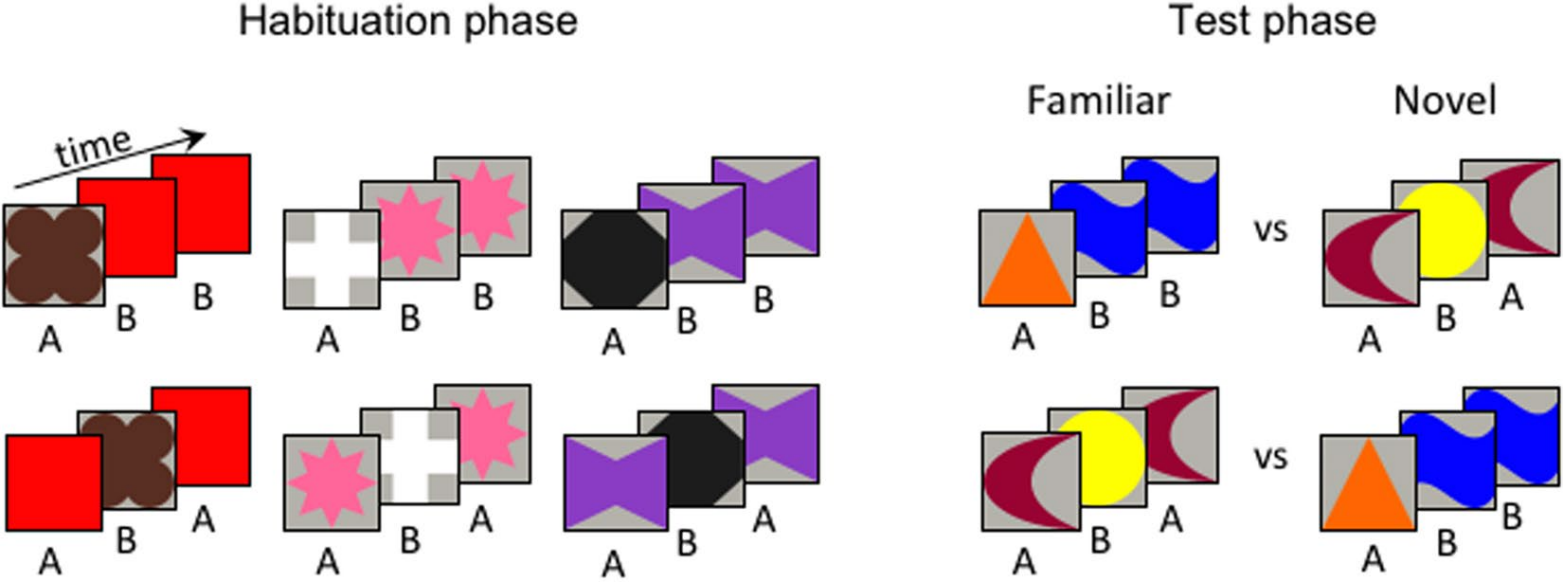
Examples of the stimuli used during the habituation and the test phases of Experiments 1 and 2. The shapes within each triplet were presented sequentially on the screen, from left to right in Experiment 1, and from right to left in Experiment 2. Half of the infants in each experiment were habituated to ABB rule-like sequences, whereas the other half were habituated to ABA rule-like sequences.

To provide a direct test for this hypothesis we presented 7-month-old infants with rule-based visual sequences of geometrical shapes (Fig. 1), manipulating the complexity of the rule (ABB vs. ABA) and the spatial orientation of the sequences (left-to-right vs. right-to-left). As already noted, 7-month-olds’ ability to represent and discriminate ordinal relations in numerical sequences is modulated by spatial information, with an advantage for a left-to-right over a right-to-left spatial orientation^24^. Similarly, it is possible that, irrespective of rule complexity, a left-to-right spatial orientation (Experiment 1) helps the extraction of serial order information defining rule-like patterns, resulting in infants’ successful learning of both the ABB and the ABA rules. In contrast, it is possible that a right-to-left presentation (Experiment 2) hinders the ability to learn even the simpler ABB rule, an ability that is otherwise functional when no directional information is provided^31^.

## Results

### Exp.1

All infants reached the habituation criterion, requiring an average of 8.20 trials (SE = 0.52) and a looking time of 93.87 seconds (s) (SE = 8.27). An ANOVA on habituation looking times with rule (ABB vs. ABA) as the between-participants factor and habituation trials (first three vs. last three) as the within-participants factor confirmed the presence of an overall significant decline in mean looking time from the first three (M = 51.08, SE = 4.46) to the last three habituation trials (M = 18.96 s, SE = 1.87), *F*(1, 30) = 111.04, *p* < 0.001, η^2^ = 0.79. No other effects or interactions were significant (*p*s > 0.2).

**Figure 2:**
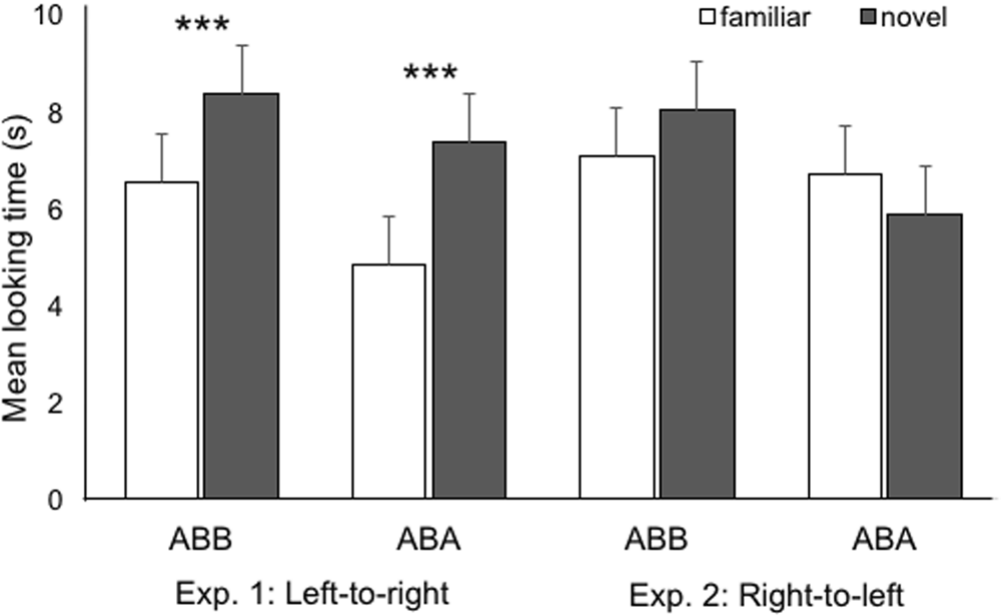
Mean looking times (±SE) to the familiar and to the novel test trials for infants presented with left-to-right oriented sequences in Experiment 1 and for infants presented with right-to-left oriented sequences in Experiment 2. Infants in Experiment 1 showed significantly longer looking times to the novel test trials than to the familiar test trials, whereas infants in Experiment 2 failed to discriminate between the two. ***p < 0.001.

To determine whether in test infants were able to discriminate the familiar from the novel rule-like patterns, an ANOVA was performed on looking times during novel and familiar test trials, with rule (ABB vs. ABA) and test order (familiar first vs. novel first) as between-participants factors, and test trial pair (first vs. second vs. third) and test trial type (novel vs. familiar) as within-participants factors. There was a main effect of test trial type, *F*(1, 28) = 40.8, *p* < 0.001, η^2^ = 0.59, as infants looked overall longer to the novel test trials (M = 7.86 s, SE = 0.69) than to the familiar ones (M = 5.67 s, SE = 0.52) (Fig. 2). There was also a main effect of test trial pair, *F*(2, 56) = 8.69, *p* = 0.001, η^2^ = 0.24, which was qualified by a Test Trial Pair × Test Trial Type × Test Order interaction, *F*(2, 56) = 4.1, *p* = 0.02, η^2^ = 0.13. Follow-up comparisons (Bonferroni corrected) revealed that infants who began the test phase with a novel trial type looked significantly longer to the first pair of test trials (M = 8.75 s, SE = 1.5) than to the second (M = 5.28 s, SE = 0.56; *p* = 0.03). No other effects or interactions were significant (*p*s > 0.1).

These results indicate that infants were able to extract and represent serially ordered information from the left-to-right oriented sequences during habituation, and to generalize it to the new sequences presented at test. Crucially, this ability was not modulated by the complexity of the rule-based pattern (ABB or ABA) embedded in the habituation sequences (*p* > 0.2).

### Exp.2

All infants reached the habituation criterion, requiring 7.69 (SE = 0.46) trials and an overall looking time of 106.19 s (SE = 8.9). A 2 (rule: ABB vs. ABA) × 2 (habituation trial: first three vs. last three) ANOVA confirmed that all infants manifested an overall significant decline in mean looking time from the first three (M = 65.17 s, SE = 7.2) to the last three habituation trials (M = 18.91 s, SE = 1.6), *F*(1, 30) = 57.84, *p* < 0.001, η^2^ = 0.66, with no variations depending on the rule embedded in the habituation sequences (*p* > 0.9). Number of trials and total looking time to habituate did not differ from those shown by infants in Experiment 1 (*p*s > 0.3).

The 4-way ANOVA on looking times at test, with rule (ABB vs. ABA) and test order (familiar first vs. novel first) as between-participants factors, and test trial pair (first vs. second vs. third) and test trial type (novel vs. familiar) as within-participants factors, revealed only a main effect of test trial pair *F*(2, 56) = 9.95, *p* < 0.001, η^2^ = 0.26, as infants’ looking times were longer in the first (M = 8.84 s, SE = 0.89) than in the second (M = 6.49 s, SE = 0.67; *p* = 0.02) and the third (M = 5.68 s, SE = 0.59; *p* = 0.001) trial pairs, indicating a weariness effect in the last test trials. Crucially, there were no main effects nor interactions involving the factors rule and test trial type (*p*s > 0.1), indicating that neither infants habituated to the simpler rule (ABB) nor those habituated to the more complex one (ABA) succeeded at discriminating the familiar from the novel test sequences (Fig. 2). These results suggest that, unlike infants tested with left-to-right sequences in Experiment 1, infants tested with right-to-left oriented sequences have not parsed serial order information delivering rule-like patterns, thus failing to learn, represent and generalize the rule embedded in habituation sequences to the newly-shaped sequences presented at test.

Overall, these findings demonstrate that the spatial orientation of the to-be-learned sequences modulates infants’ rule learning abilities, with an advantage for a left-to-right over a right-to-left spatial orientation similar to that shown during learning of numerical order (de Hevia *et al*.^24^). This conclusion was further confirmed by a 5-way ANOVA on looking times toward novel and familiar test trials with spatial orientation (left-to-right vs. right-to-left) as an additional between-participants factor: in addition to significant main effects of test trial type, *F*(1, 56) = 8.6, *p* = 0.005, η^2^ = 0.13, and test trial pair, *F*(2, 112) = 18.53, *p* < 0.001, η^2^ = 0.25, there was a significant Test Trial Type × Spatial Orientation interaction, *F*(1, 56) = 5.36, *p* = 0.024, η^2^ = 0.087.

## Discussion

The present study provides the first evidence that spatial orientation does impact preverbal infants’ perception and learning of ordered information in (non-numerical) visual sequences where items’ order delivers rule-like patterns. Seven-month-old infants in the current study were able to extract rule-like patterns specified by adjacent-late (ABB) and non-adjacent (ABA) repetitions of one element when sequences were presented from left to right, but failed with a right-to-left spatial orientation. This finding indicates that infants’ rule learning abilities are strongly impacted by directional spatial information. Earlier studies have shown that, in the absence of spatial information (i.e., when visual sequences are presented centrally on the screen), infants at this same age can extract and learn the ABB rule but fail with the more complex ABA rule with elements represented by visual geometric shapes^31^. Therefore, infants’ success at learning the ABA rule in the present study (Experiment 1) demonstrates that a left-to-right orientation boosts infants’ rule learning, whereas infants’ failure at learning the ABB rule (Experiment 2) demonstrates that a right-to-left orientation hinders this ability. These findings provide the first evidence that preverbal infants spontaneously relate ordered information to left-to-right oriented spatial codes for non-ordered information also, and not only for intrinsically ordered information (i.e., refs 23, 24, 27).

Spatial-temporal congruency effects similar to the one reported here have been described in adults, who show more accurate recall of temporal ordinal information stored in working memory when the spatial layout of the stimuli is congruent with the directionality of the subjects’ reading/writing experience (i.e., from left to right in English^19^). These findings have been interpreted as evidence of shared representations for both temporally and spatially conveyed ordered information, supporting the view that temporary position-space associations are constructed in working memory during task execution^16, 18, 22^. Without denying the modulating role of formal education and symbolic knowledge in shaping the direction of the spatial-order association, our results suggest that humans are prone to represent ordered information along a left-to-right spatial continuum since early stages of development. The non-verbal nature of the stimuli used in the current study, along with the participants’ young age, allows to conclude that the involvement of space in order processing is not limited to the verbal domain, but reflects a property of serial order working memory that is independent of the modality of the to-be-remembered items^34, 35^.

Nonetheless, if formal education and symbolic knowledge do not play a critical role in establishing this early mapping of ordered information into oriented spatial codes, what could be the determinants of its appearance? One possibility is that the mapping derives from early exposure to culturally-driven routines, such as observing how adults read books, draw pictures and order items in space, which in turns provides infants with implicit, directionally relevant experience (see refs 36 and 37). These early directional cues might shape the direction of infants’ spatial representation of order depending on the dominant direction of their cultural environment. Alternatively, the emergence of a left-to-right spatial organization of ordered dimensions during the first months of life might be rooted in biologically-determined neural constraints in the human brain. Indeed, the right hemisphere is dominant in visuo-spatial task^38^, and it has recently been proposed that early temporal asymmetries in hemispheric maturation, with a temporal advantage for the right over the left hemisphere^39^, may determine a leftward asymmetrical exploration of visual space that would constrain the structure of infant’s representational space^26^. The possibility of a link between a right hemispheric dominance and a left-to-right representation of ordinal information is also suggested by studies with non-human animals^40–42^.

However, these two hypotheses might not be mutually exclusive, as in the earliest stages of development experience with cultural-based attentional routines might combine with a right hemispheric dominance in shaping the directionality of the order-space mapping^26^. On this account, the representation of ordinal information into a directional spatial code might emerge from the interaction between early culturally-driven directional cues and biologically-determined attentional biases in exploring the external space. Future research may investigate further for the presence of such constraints by exploring the neural underpinnings of preverbal infants’ representation of serial order, as well as by testing newborn infants and infants growing up in cultures with different reading and writing systems.

## Methods

### Participants

A total of 64 7-month-old infants (34 females, mean age = 7 months 17 days, range = 7 months 2 days to 8 months 1 day), randomly assigned to Experiment 1 or Experiment 2, participated in this study. All participants were healthy and full-term, and they were all Caucasian. Three additional infants were excluded from the final sample because of fussiness. Participants were recruited via a written invitation that was sent to parents based on birth records provided by neighboring cities. The protocol was carried out in accordance with the ethical standards of the Declaration of Helsinki (BMJ 1991; 302: 1194) and approved by the Ethical Committee of the University of Milano-Bicocca. Parents gave written informed consent for their infants’ participation.

### Stimuli and procedure

Twelve unique colored shapes were organized into ABB or ABA triplets. Following Saffran *et al*.^32^, eight unique shapes were presented during habituation, and four different unique shapes were presented during the test phase. When viewed from a distance of 60 cm, each shape was embedded in a virtual square of 10° × 10° visual angle. For the habituation triplets, four unique shapes were assigned to the A group and four to the B group. The A and B images were randomly combined by the software to generate 16 different ABA triads (i.e., a shape A was followed by a different shape B, which was in turn followed by the shape A) and 16 different ABB triads. Four novel unique shapes made up the triplets during the test phase, two assigned to the group A and two assigned to the group B. A left-to-right (Experiment 1) or a right-to-left (Experiment 2) sequential presentation of the images within each triad was used (Fig. 1). For the left-to-right sequences, the first image was displayed on the left side of the monitor for 330 ms, the second image was displayed in the middle of the monitor for 330 ms, then the third image was displayed on the right side of the monitor for 830 ms. The distance between the center of each figure was 16° of visual angle. For the right-to-left sequences, the three images of the triad were presented from right to left. A blank screen (500 ms) separated the triad presentations on each trial. Half of the infants was randomly assigned to the ABB habituation condition, the other half to the ABA habituation condition.

An infant-controlled habituation procedure was used. A cartoon animated image associated with varying sounds served as an attention getter before the trial began. As soon as the infant fixated the screen the experimenter turned off the cartoon and began a trial. Each trial consisted of triads of images, presented in a random order, organized in either the ABB or ABA pattern. The experimenter recorded infant’s fixation by holding the mouse button whenever the infant fixated on the stimulus. Each trial continued until the infant looked continuously for a minimum of 500 ms and ended when the infant looked away for 2 consecutive seconds or looked for a maximum of 60 s. The habituation phase ended when the infant saw a maximum of 25 trials - after Saffran *et al*.^32^ - or met the habituation criterion, which was defined as a 50% decline in looking time on three consecutive trials, relative to the looking time on the first three trials^43^. Following the habituation phase, infants were given 6 test trials in which ABA and ABB triads, composed by images that differed from those showed during habituation, were presented alternately, each for three times. The order of presentation (i.e., novel or familiar first) was counterbalanced among infants. Looking time (s) towards novel and familiar patterns was considered as the dependent variable.

### Apparatus

Each infant was tested while sitting on an infant seat or on the parent’s lap and positioned at a distance of approximately 60 cm from the monitor where the stimuli were presented (24” screen size, 1920 × 1200 pixel resolution). The whole experiment was recorded through a video-camera, hidden over the screen, which fed into a TV monitor and a digital video recorder, both located outside the testing cabin. The TV monitor displayed the live image of the infant’s face to allow the online coding of the infant’s looking times through the E-Prime program by the experimenter, who was blind to the condition to which the infant was assigned.

The image of the infant’s face was also recorded via a Mini-DV digital recorder for a frame-by-frame offline coding of looking times during test trials. For half of the infants (N = 30) looking times were coded offline by a second independent observer who was blind to the experimental condition. Inter-observer agreement (Pearson correlation) between the two observers (i.e., the one who coded the data online and the one who coded from digital recording), as computed on total fixation times during test trials, was *r* = 0.97, *p* < 0.001.
